# Survival Analysis Part III: Multivariate data analysis – choosing a model and assessing its adequacy and fit

**DOI:** 10.1038/sj.bjc.6601120

**Published:** 2003-08-12

**Authors:** M J Bradburn, T G Clark, S B Love, D G Altman

**Affiliations:** 1 Cancer Research UK/NHS Centre for Statistics in Medicine, Institute of Health Sciences, Old Road, Oxford OX3 7LF, UK

**Keywords:** survival analysis, Cox model, AFT model, model checking, choice of coavriates, goodness of fit

## INTRODUCTION

In this series of papers, we have described a selection of statistical methods used for the initial analysis of survival time data (Clark *et al*, 2003), and introduced a selection of more advanced methods to deal with the situation where several factors impact on the survival process (Bradburn *et al*, 2003). The latter paper focused on proportional hazards (PH) and accelerated failure time (AFT) models, and we continue the series by demonstrating the application of these models in more detail. Whereas the focus of the previous paper was to outline the purpose and interpretation of statistical models for survival analysis, we concentrate here on approaches with which to undertake the actual modelling process. In other words, the aim of this paper is to promote the correct use of the models that have been suggested for the analysis of survival data.

When used inappropriately, statistical models may give rise to misleading conclusions. Checking that a given model is an appropriate representation of the data is therefore an important step. Unfortunately, this is a complicated exercise, and one that has formed the subject of entire books. Here, we aim to present an overview of some of the major issues involved, and to provide general guidance when developing and applying a statistical model. We start by presenting approaches that can be used to ensure that the correct factors have been chosen. Following this, we describe some approaches that will help decide whether the statistical model adequately reflects the survivor patterns observed. Lastly, we describe methods to establish the validity of any assumptions the modelling process makes. We will illustrate each using the two example datasets (a lung cancer trial and an ovarian cancer dataset) that were introduced in the previous papers (Bradburn *et al*, 2003; Clark *et al*, 2003).

## CHOICE OF COVARIATES

The covariates that we consider here are fixed, that is, known at baseline or entry to the study. The handling of covariates that change values over time (e.g. white blood cell count as measured at different time points) will be described in the subsequent paper in this series.

### Sample size considerations

It is implicitly assumed that the subjects in a study are representative of a wider population to enable the study aims to be addressed. Another important requirement is to have data from an adequate number of subjects. Any estimate based on a small number of individuals will be less reliable than one based on a larger number, and when multivariate models are fitted to small datasets, the estimated impact of the covariates is too imprecise to give reliable answers. The use of variable selection procedures as described below is especially problematic with such data, and often leads to overoptimistic results. Finally, smaller data sets may not have sufficient power to detect a covariate that has a significant impact on survival.

The power (and indeed in some cases validity) of a survival analysis is related to the number of events rather than the number of participants. Simulation work has suggested that at least 10 events need to be observed for each covariate considered, and anything less will lead to problems, for example, the regression coefficients become biased (Peduzzi *et al*, 1995). In the ovarian study, there were 550 deaths and 11 covariates for the five prognostic factors, implying 50 events per covariate. In the liver cancer trial with 114 events, a full model of 11 covariates has approximately 10 events per covariate.

For prospective studies, several books (e.g. Machin *et al*, 1998) and software packages (e.g. nQuery, power and precision) are available to assist the calculation of adequate sample sizes, and many general purpose statistical packages also perform such calculations.

### The aim of the study influences the choice of covariates

Before embarking on any statistical modelling, it is helpful to be clear as to why the multivariate model is to be fitted. The models we have presented have the considerable advantage of being able to handle several factors simultaneously, but the choice of which to incorporate lies with the analyst. This choice depends on the study aims. We suggest three possible scenarios as to why a study may use a multivariate model, and deal with each in turn.

#### (a) A single factor is under investigation for its association with survival, but several other factors exist

The rationale of such a study is to perform a specific test of one factor. This scenario may arise in a randomised controlled trial, such as the lung cancer example, where the aim is to decide whether a new treatment prolongs survival, but also to adjust for prognostic factors that may or may not be equally matched between treatment groups. Another situation occurs where an association between a marker and patient survival is being assessed. In either case, any terms that are of potential importance could be incorporated whether significant or not, depending on the adequacy of the sample size. All of the covariates (other than the one of primary interest) are essentially ‘nuisance’ factors that are considered only to ensure they have been taken due account for in assessing the importance of the (prespecified) factor under investigation. Less important covariates may be removed.

#### (b) A collection of factors of known relevance are under investigation for their ability to predict survival

This arises when one wishes to assess the individual importance of a series of factors, and/or to attempt to build a model that helps predict patient survival. In such cases, the simplest strategy is to attempt to model all covariates, obtain effect sizes and gauge how well the model predicts survival. It may be desirable to remove factors from the model for simplicity, provided this does not compromise the predictive ability of the model. Statistical significance alone is an insufficient measure of assessing the extent to which a covariate can predict survival. Methods that may be used for this evaluation are given in the final paper of this series.

#### (c) Where a collection of factors are under investigation for their potential association with survival, possibly with additional known factors

Such studies are more ‘exploratory’ in nature, and the aim is to identify quantities of potential importance for further investigation. Here it is often desired to reduce the number of covariates in the model by excluding those that are not statistically significant and thus concentrate only on ‘potentially interesting’ ones for future research. Care must be exercised when several covariates are investigated, as the false-positive rate (or the chance of finding a spurious effect) increases with each additional test.

This selection of scenarios is far from exhaustive, and in practice a study may combine all of the above types. The ovarian study is a combination of (b) and (c).

### *Approaches to adding or removing covariates*

Common choices for model building focus on ‘semiautomated’ methods such as stepwise selection, but other approaches exist. Models that are based purely on statistical significance may not be clinically meaningful. Henderson and Velleman (1981) state this simply: ‘The data analyst knows more than the computer’, and appropriate use of this knowledge should be incorporated into the analysis. We recommend that the choice of covariates should be verified by a degree of hands-on modelling, where terms are added or removed in a logical order rather than solely according to statistical significance.

We illustrate some straightforward approaches to the choice of covariates in the two example datasets used in this paper. In the final paper of this series, we will outline the rationale behind semiautomated methods (together with their limitations) and give further advice on hands-on modelling.

### Selecting covariates for the lung cancer trial

As stated before, the lung cancer trial as presented in the earlier paper is an example of scenario (a). The table of coefficients for the full AFT multivariate model was presented in the previous paper (Bradburn *et al*, 2003). A simpler model would be to consider just the performance status, cell type and treatment covariates. Removing the remaining covariates reduces the model likelihood, but not to a significant degree (*χ*^2^=3.34 on 8 degrees of freedom; *P*=0.91). The new time ratios, confidence intervals and *P*-values are presented in Table 1

**Table 1:** Generalised gamma AFT model applied to the lung cancer data

**Covariate**	**Coefficient (*b*_*i*_)**	**TR [exp(*b*_*i*_)]**	**95% CI**	***P*-value**
Treatment (RT+CAP *vs* RT alone)	0.640	1.90	(1.23–2.93)	0.004
				
Cell type (squamous *vs* nonsquamous)	0.536	1.71	(1.08–2.71)	0.02
				
Performance status (8–10 *vs* 5–7)	0.765	2.15	(1.09–4.24)	0.03

TR=time ratio; CI=confidence Interval; RT=radiotherapy; CAP=cytoxan, doxorubicin and platinum-based chemotherapy.

. They are virtually unchanged from the previous analysis, and thus the earlier conclusions remain the same.

### Selecting covariates for the ovarian cancer database

As stated previously, the analysis of the ovarian cancer database (as described in the previous paper) could be considered as a mixture of scenarios (b) and (c). However, as the database is large and the aim is to derive a prognostic model, we will focus on (b). We consider five covariates here, all of which were measured at diagnosis: FIGO stage (an ordinal covariate taking values 1, 2, 3 or 4), histology (with seven possible subtypes), grade, ascites (yes/no) and patient age.

In this analysis, all the covariates were included yielding the model presented in Table 2

**Table 2:** Cox model applied to the ovarian data

**Covariate**	**Coefficient (*b*_*i*_)**	**HR [exp(*b*_*i*_)]**	**95% CI**	***P*-value**
FIGO stage	0.731	2.08	(1.82–2.37)	<0.001
				
*Histology*				<0.001
Serous	(0.000)	(1.00)		
Mucinous	−0.422	0.66	(0.50–0.85)	
Endometroid	0.198	1.22	(0.80–1.85)	
Clear cell	0.342	1.41	(0.99–2.00)	
Adenocarcinoma	0.501	1.65	(0.91–2.99)	
Undifferentiated	0.746	2.11	(1.03–4.29)	
Mixed mesodermal	0.789	2.20	(1.45–3.35)	
				
*Grade*				<0.001
1	(0.000)	(1.00)		
2	0.885	2.42	(1.40–4.19)	
3	0.885	2.42	(1.40–4.18)	
				
Absence of ascites	−0.396	0.67	(0.54–0.84)	<0.001
Age (per 5-year increase)	0.133	1.14	(1.09–1.19)	<0.001

HR=hazard ratio; CI=confidence interval.

. Advanced FIGO stage, higher grade, presence of ascites and increased age all impaired survival to varying degrees. The mucinous and serous histology types had a better prognosis, and undifferentiated and mixed mesodermal a lesser one. No grade–histology interactions were included in the final model, either due to insufficient numbers of patients to allow meaningful modelling (e.g. clear cell, mixed mesodermal, adenocarcinoma or undifferentiated), or for statistical insignificance (the remainder). In fact no second-order interaction or nonlinearity was detected.

If this model were to be used for the purpose of predicting future survival patterns, it is appropriate to ensure that the effect sizes are robust. One approach is to use bootstrap sampling, which involves randomly resampling the data and fitting the model to these modified datasets (Clark and Altman, 2002). These produce a series of effect sizes that should be similar to those derived from the original data if the model is sufficiently stable, and indeed do so here.

## ASSESSING THE ADEQUACY OF A MODEL

Regardless of which type of model is fitted and how the variables are selected to be in the model, it is important to evaluate how well the model represents the data. A survival model is adequate if it represents the survival patterns in the data to an acceptable degree. This aspect of a model is known as *goodness of fit*. For example, if a given group of patients have a poor (or good) prognosis, then the model should predict this group to have that outcome. In practice, the issues in choosing the most appropriate type of model and the most appropriate covariates are heavily related, and the adequacy of a model may be assessed in several ways. In this section, we discuss methods to verify fit that are common across all survival models, before describing approaches specific to different model types. We will use the ovarian database example to demonstrate these checks.

### Residuals from survival models

Residuals are a useful method for checking the fit of a statistical model. Essentially, they are the difference between an observed and a model-predicted quantity, with large or systematic differences between the two indicative of a poor model. Several residuals have been proposed, but unfortunately most are rather difficult to understand in the context of survival analyses due to censoring (Collett, 1994). In general, the residuals are skewed and need to have smoothing functions (e.g. Kernel smoother) applied to aid interpretation. Nevertheless, the graphical displays suggested in Table 3

**Table 3:** Suggested plots for residual-based diagnostics

***Y*-axis**	***X*-axis**	**Potential implication**	**Suggested remedy**
Martingale residual	Any omitted covariate	Covariate excluded wrongly	Refit model with covariate included
Martingale residual	Any included covariate	Covariate modelled incorrectly (e.g. nonlinear effect)	Fit nonlinear term (e.g. a squared term)
Martingale residual	Date of enrolment in study	Evidence of temporal effect	Incorporate time of entry as a covariate
Deviance residual	Survival time, log(survival time) or ranks of survival time	Model fails to predict consistently for all survival times	Fit time-dependent PH model or consider using a different (i.e. non-PH) model
Deviance residual	Subject identifier	Individual is an outlier	(1) Check if the data are correct
			(2) Refit model with individual removed. If effect sizes alter substantially, consider removing individual altogether
Scaled Schoenfeld residual	Survival time, log(survival time) or ranks of survival time	Non-PH	Fit time-dependent PH model or consider using a different (i.e. non-PH) model

PH=proportional hazards. All of the above *X*–*Y* plots should give rise to a plot evenly scattered along a horizontal line that displays no trend. The possible implications where this does not occur and suggested remedies are presented.

(with appropriate smoothing as required) should all give rise to an evenly scattered horizontal band and display no obvious trend (e.g. no slope). If a trend in these plots is apparent, it should be investigated, perhaps using the method suggested in Table 3. Overall model adequacy may be assessed by use of Cox-Snell residuals (Collett, 1994).

### Residual plots for the ovarian cancer data set

Figures 1A

**Figure 1:**
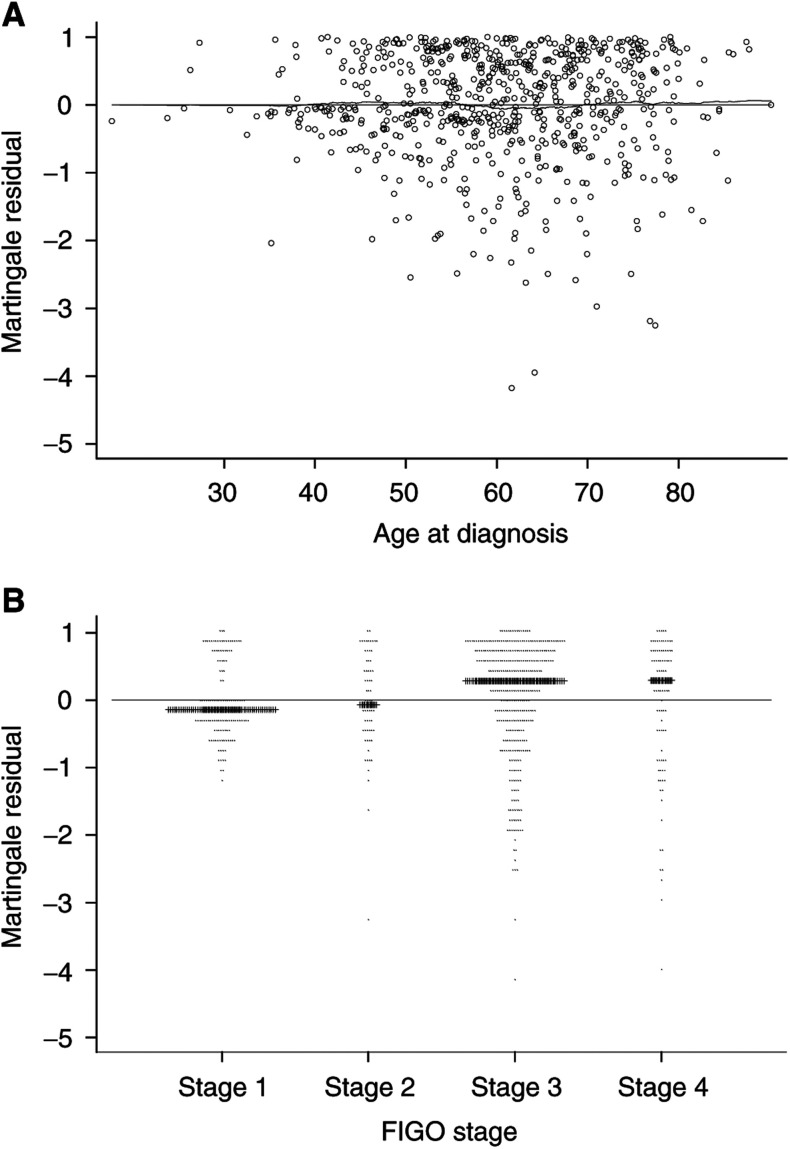
Martingale residuals plotted against (**A**) patient age and (**B**) FIGO stage; median for each stage is denoted by a horizontal line.

illustrates a plot of the Martingale residuals against the patient's age, with a Kernel smoother marked as the dashed line. Figure 1B shows the Martingale residuals plotted against FIGO stage, with the median within each stage represented by the solid bar. Both FIGO and age were modelled as linear effects. If FIGO or age had been wrongly excluded or modelled incorrectly (i.e. nonlinear), the figures should display a trend other than a strictly horizontal line. The age residual plot shows no evidence of a trend. Although there appears to be evidence of a trend in the FIGO plot, the inclusion of this covariate as a categorical covariate fails to improve the fit to a significant degree. Thus, we may be reasonably confident that the model is adequate for both covariates.

### Identifying the correct parametric model

When fitting a fully parametric model, the survival times are assumed to follow a statistical distribution. Several different distributions have been proposed, and the identification of a suitable one is a crucial step. The most obvious distinguishing feature between parametric models is in the shape of the hazard they assume the data follow. The Weibull and Gompertz distributions are appropriate when the hazard is always increasing or decreasing; the Log-Logistic may be used where the hazard either rises to a peak and then decreases or always decreases; the Log-Normal and Generalised Gamma models are preferable when the hazard rises to a peak before decreasing. In the Exponential model, the hazard is assumed to be constant over time. The actual shapes of these distributions (e.g. the point in time at which a hazard ‘peaks’ or the gradient at which it increases/decreases) depend on *ancillary* parameters that are also estimated from the data. For example, when using the Weibull distribution, the hazard function, *h*(*t*), is *λs*(*λt*)^*s*−1^. In this case, the shape (*s*) and scale (*λ*) parameters are the ancillary parameters to be estimated (see Figure 1 in the previous paper Bradburn *et al*, 2003).

If the shape of the disease hazard is known to be different from that of a particular distribution, then the data should not be analysed with this parametric model. For example, consider the hazard for overall survival after cancer diagnosis. The hazard is rarely constant, thus ruling out an Exponential distribution. In some cases, the hazard rises sharply (due to treatment deaths) before tailing off, which would also rule out the Weibull. An informal assessment of a parametric model's appropriateness may be made via plotting the (smoothed) empirical hazard or cumulative hazard against those estimated by the model, or by log(−log(survival)) plots which are discussed later. Akaike's Information Criterion (AIC) (Akaike, 1974), a statistic that trades off a model's likelihood against its complexity, may also be used when comparing the viability of different parametric models. The AIC of a model may be defined as


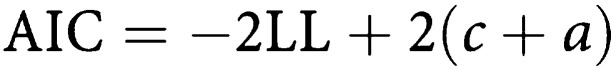


where LL is the logarithm of the model likelihood (*log-likelihood*), *c* is the number of *covariates* and *s* the number of ancillary parameters (e.g. 2 in the case of the Weibull; *λ* and *s*). A *lower* value of the AIC suggests a better model. Note, however, that the likelihood computed in a Cox model is a partial likelihood, and so it is not possible to compare Cox PH models to fully parametric ones in this manner.

In the PH framework, it may be clear that none of the parametric models suggested here or elsewhere adequately capture the distributional form of the data. In such cases, the more flexible Cox model is the obvious choice. Commonly used parametric models in the AFT framework are arguably more flexible than those available in the PH framework, and so fitting a parametric AFT model is another option.

### Overall goodness-of-fit tests

A simple test for the model adequacy is to compare the overall (Kaplan–Meier) survival curve to the model-based predicted survival and, ideally, for any group of patients the two should be close, if not identical. Hosmer and Lemeshow (1999) suggest using a more formal measure of fit based on comparing observed and expected events in different *risk groups* as defined by the model. Specifically, the predicted risk or prognostic index (PI) from a model consisting of covariates *x*_1_, *x*_2_, …, *x_p_* with estimated coefficients *b*_1_, *b*_2_, …, *b_p_*, respectively, is


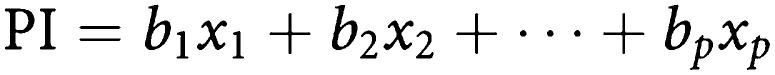


PI is calculated for each patient. Risk groups are constructed by categorising the (ranked) PIs, for example, three risk groups can be created using the highest, middle and lowest tertiles of PI. A score test is then applied to the differences between the observed and expected events in the risk groups. A simple approximation to this calculation may be obtained by adding the risk groups as a series of covariates to the survival model itself. A significant improvement in the model likelihood suggests that the original covariates form an insufficient model for the data.

### Assessing overall goodness of fit on the ovarian cancer data

The predicted survival curves for the ovarian cancer model are potentially misleading. Several factors are associated with length of survival, and some are also related to (or correlated with) each other (e.g. histology and stage). Predicted survival curves for each histological group may be estimated by fixing all other covariates at their mean values. However, this approach will give an estimate of survival that is different to those observed in the data because correlations are ignored. The test of Hosmer and Lemeshow (1999) is more useful here. The patients are split into ten risk groups, with the proportion of deaths in each ranging from 10% in the best prognosis group to 94% in the worst. The approximate score test, derived from adding nine covariates to the model, produced no evidence of a poor fit (likelihood ratio test *χ*^2^=7.84 on 9 degrees of freedom, *P*=0.55).

## ASSESSING WHETHER PH IS APPROPRIATE

The PH assumption, that is, the hazards are proportional (and not overlapping) at all points in time, should be verified. An obvious approach is to plot the hazard in each group, but this is of limited use. The empirical hazard function is generally not well estimated, and instead the cumulative hazard is generally preferred to assess the PH assumption. If a PH model is valid, a plot of the logarithm of the cumulative hazard function in each group against the logarithm of time should give rise to lines that are parallel. Continuous variables need to be categorised into groups. The plot described is also known as the log(−log(survival)) plot, as the cumulative hazard is equal to the negative logarithm of the survival proportion. This approach requires a subjective assessment. Unfortunately, convergent or divergent lines may be due to either a lack of proportionality or to the omission of an important covariate. In practice, it is not known which, but this phenomenon suggests an inadequate model. On the other hand, parallel lines suggest that models assuming PHs may be suitable. In the case of fitting a Weibull or an Exponential parametric model, the lines should be parallel and straight.

Several formal statistical tests have been proposed for assessment of proportionality of hazards. A simulation study by Ng'andu (1997) described and compared several tests in the Cox PH framework, and concluded that the (weighted) scaled Schoenfeld residuals test (Grambsch and Therneau, 1994), the linear correlation test (Harrell, 1986) and the time-dependent covariate test (Cox, 1972) were the most powerful diagnostic tools for proportionality. The first two of these test for an association between residuals and time (evidence of which indicates a bad fit), and the third tests whether the effect (coefficient) of a covariate changes with time (i.e. nonconstant hazard ratio). This latter method is appealing as it not only detects nonproportionality, but allows it to be modelled validly. An alternative is to fit a *stratified* model, wherein a covariate that displays nonproportionality is modelled without the constraint of proportionality. Such a covariate must obviously be categorical (or be categorised), but more importantly has no estimated effect size provided when forming the strata of a stratified model, and thus is suitable only for covariates that are not of primary interest. Abandoning the PH approach in favour of some other model is clearly another option.

### Assessing the appropriateness of PH for the ovarian cancer data

**Figure 2:**
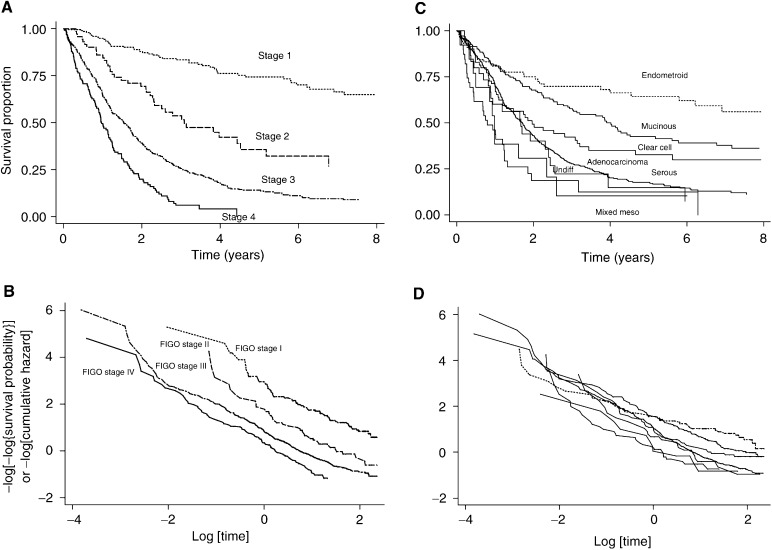
(**A**) Survival according to FIGO stage. (**B**) Log(−log(survival)) for FIGO stage. (**c**) Survival according to histology. (**D**) Log(−log(survival)) for histology. The endometroid group is shown by the dotted line.

The Kaplan–Meier survival curves and log(−log(survival)) *vs* log(time) plots are shown for FIGO stage and histology in Figure 2A–D

. The log(–log(survival)) plot for FIGO stage gave rise to reasonably parallel lines and therefore suggests proportionality. However, in the case of the histology, this appears to be violated. In particular, the prognosis for the endometroid group sits in the middle of all the groups in the first year but improves thereafter. A similar feature was apparent for the presence of ascites, where the initial detrimental effect becomes less important with time (data not shown). The (weighted) scaled Schoenfeld residuals test suggested significant overall nonproportionality (*P*=0.05), as did the time-dependent covariate tests for these terms. Therefore, despite other aspects of this model appearing adequate, the assumption of proportionality appears to be violated.

Nevertheless, we can still use a Cox PH model with time-dependent covariates implemented, which is a model that includes interaction terms between the covariates and (log) time, and thus allows the effect of the relevant covariates to change with time. Table 4

**Table 4:** The Cox model applied to the ovarian data, with a time dependency added to ascites and endometroid terms

**Covariate**	**Coefficient (*b*_*i*_)**	**HR [exp(*b*_*i*_)]**	**95% CI**	***P*-value**
FIGO	0.734	2.09	(1.83–2.38)	<0.001
				
*Histology*				<0.001
Serous	(0.000)	(1.00)		
Mucinous	−0.432	0.65	(0.50–0.85)	
Clear cell	0.344	1.41	(0.99–2.01)	
Adenocarcinoma	0.494	1.64	(0.91–2.96)	
Undifferentiated	0.769	2.16	(1.06–4.40)	
Mixed mesodermal	0.825	2.28	(1.50–3.47)	
Endometroid	0.312	1.37	(0.90–2.07)	
Endometroid × log(time)	−0.500	0.61	(0.45–0.82)	0.001
				
*Grade*				<0.001
1	(0.000)	(1.00)		
2	0.826	2.28	(1.32–3.95)	
3	0.843	2.32	(1.35–4.00)	
				
*Absence of ascites*	−0.466	0.63	(0.50–0.80)	<0.001
Ascites × log(time)	0.233	1.26	(1.01–1.58)	0.04
				
Age (per 5-year increase)	0.134	1.14	(1.09–1.20)	<0.001

R=hazard ratio; CI=confidence interval.

shows the amended model that now allows the effects to vary with time. The time-dependent terms suggest that the absence of ascites and endometroid histology have effects that diminish (the hazard ratios tend towards 1) with time. For example, the absence of ascites is judged to have a hazard ratio of exp(−0.466+0.233 × log(2))=0.74 at 2 years but exp(−0.466+ 0.233 × log(5))=0.91 at 5 years.

## ASSESSING WHETHER AN AFT MODEL IS ADEQUATE

In the AFT model, the survival proportion in one group at any time *t* is equal to the survival proportion in the second at time *ϕt*, where *ϕ* is constant. Therefore, a Quantile–Quantile (Q–Q) plot of the times of survival percentiles should lie on a straight line of slope *ϕ* that passes through (0, 0). As with the log(−log(survival)) plot in PH models, this is a useful but limited approach as departures from linearity could be due to the AFT model being inappropriate or that one or more important covariates have been omitted. The methods of stratification or modelling with time-dependent covariates suggested in the PH section may be applied here as well.

### The lung cancer trial data

We assessed the adequacy of the Generalised Gamma and four other parametric models (each with all covariates included) and present their AIC values in Table 5

**Table 5:** Akaike Information Criterion (AIC) of five different distributions fitted to the full model

**Model**	**Log likelihood (LL)**	**No. of covariates (*c*)**	**No. of ancillary parameters (*a*)**	**AIC**
Exponential	−259.28	11	1	542.55
Weibull	−253.21	11	2	532.41
Log-Normal	−238.22	11	2	502.44
Log-Logistic	−236.33	11	2	498.65
Generalised Gamma	−235.79	11	3	499.58

AIC=−2LL+2(*c*+*a*).

**Figure 3:**
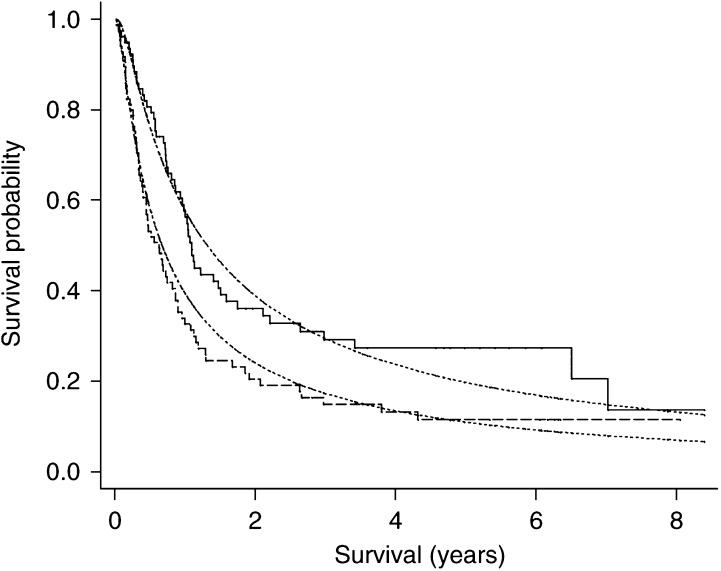
Kaplan–Meier survival probabilities for patients treated by RT+CAP (solid line) and RT alone (dashed line). The respective predicted survival proportions of a generalised gamma multivariate model are given by the faint dotted lines for grouped mean covariates. RT=radiotherapy, CAP=cytoxan, doxorubicin and platinum-based chemotherapy.

**Figure 4:**
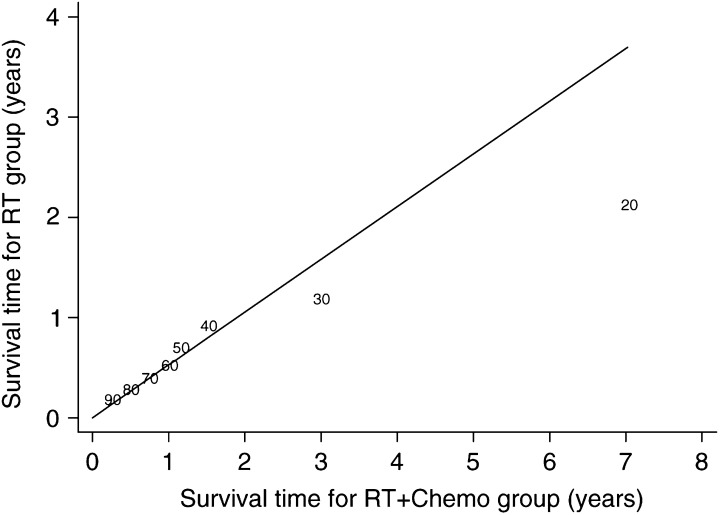
Q–Q plot (percentiles of survival distribution) for patients RT+CAP against those with RT only. The plot symbols are the survival percentiles^*^ and the slope corresponds to the value of the time ratio (=1/1.90). ^*^ The 10th percentile is omitted: 4.9 and 11.4 years for RT and RT+CAP respectively, RT=radiotherapy, CAP=cytoxan, doxorubicin and platinum-based chemotherapy.

. The Generalised Gamma model has a higher log-likelihood than the other models and a lower AIC, indicating that this distribution may be the most accurate. To check for excluded covariates, the Martingale residuals were plotted against potential model terms as before. None of these plots suggested that a covariate was incorrectly omitted. Figure 3

gives the predicted observed survival curves together with the predicted survival under a Generalised Gamma model; for each treatment group, the Karnofsky performance status and cell type were fixed at their mean values. The medium-term survival is not as well fitted by the model but is tolerably close. The long-term survival is also less well estimated, but because few patients survive this length of time the estimated survival is imprecise and so this does not cause grounds for concern. The survival times for the 10th, 20th, …, 90th survival percentiles for each treatment group are plotted as a Q–Q plot in Figure 4

and, again, apart from the later times seem to fit adequately.

## DISCUSSION

This paper has sought to demonstrate the models introduced in the previous paper in this series (Bradburn *et al*, 2003), to offer practical advice on how to select a method that represents the data fairly, and how to present and interpret it. Good modelling of survival data is not a straightforward exercise, and it is not possible to suggest an ‘off the peg’ solution. Before starting the process of deciding which (if any) of the models suggested is most suitable for an individual dataset, the important question of why the model should be fitted needs to be considered. The answer should inform the modelling process. Although it is possible to choose a model from those suggested that is optimal from a purely statistical point of view (e.g. goodness-of-fit measures), nonstatistical considerations should to be taken into account. The choice of model and of covariates therein should, in general, be suggested from experience and based on the specific question under investigation. However, good nonstatistical reasons informing model choice should not override good statistical reasons for not choosing that model. The diagnostics (e.g. residuals) for the different models may be difficult to interpret, but they will give an indication of whether modelling assumptions hold and, ultimately, should be considered when model building.

In some cases, all of the models mentioned above may not be wholly appropriate either for modelling the data or answering the relevant question. Consider an example where the time between treatment and possible multiple cancer relapse is to be investigated. The methods introduced assume one survival time (culminating in one type of event), but we may be dealing with patients who have one or more relapses of different type or levels. In the final paper of this series, we introduce models that extend the types of models described here to incorporate recurrent events. We also present approaches to modelling continuous covariates in a nonlinear fashion, validating models and discuss alternatives when fundamental censoring assumptions do not hold.
